# SMARCA4 inactivating mutations cause concomitant Coffin–Siris syndrome, microphthalmia and small‐cell carcinoma of the ovary hypercalcaemic type

**DOI:** 10.1002/path.4926

**Published:** 2017-07-25

**Authors:** Edoardo Errichiello, Noor Mustafa, Annalisa Vetro, Lucia Dora Notarangelo, Hugo de Jonge, Berardo Rinaldi, Debora Vergani, Sabrina Rita Giglio, Patrizia Morbini, Orsetta Zuffardi

**Affiliations:** ^1^ Department of Molecular Medicine University of Pavia Pavia Italy; ^2^ Unit of Paediatric Oncology Spedali Civili di Brescia Brescia Italy; ^3^ Department of Biomedical Experimental and Clinical Sciences ‘Mario Serio’ Florence Italy; ^4^ Medical Genetic Unit Meyer Children's University Hospital Florence Italy

**Keywords:** SMARCA4/BRG1, Coffin–Siris syndrome (CSS), microphthalmia, intellectual disability, small‐cell carcinoma of the ovary hypercalcaemic type (SCCOHT), SWI/SNF complex, chromatin remodelling factors, haploinsufficiency, nonsense‐mediated mRNA decay (NMD), whole‐exome sequencing (WES)

## Abstract

SMARCA4 chromatin remodelling factor is mutated in 11% of Coffin–Siris syndrome (CSS) patients and in almost all small‐cell carcinoma of the ovary hypercalcaemic type (SCCOHT) tumours. Missense mutations with gain‐of‐function or dominant‐negative effects are associated with CSS, whereas inactivating mutations, leading to loss of SMARCA4 expression, have been exclusively found in SCCOHT. We applied whole‐exome sequencing to study a 15‐year‐old patient with mild CSS who concomitantly developed SCCOHT at age 13 years. Interestingly, our patient also showed congenital microphthalmia, which has never previously been reported in CSS patients. We detected a de novo germline heterozygous nonsense mutation in exon 19 of SMARCA4 (c.2935C > T;p.Arg979*), and a somatic frameshift mutation in exon 6 (c.1236_1236delC;p.Gln413Argfs*88), causing complete loss of SMARCA4 immunostaining in the tumour. The immunohistochemical findings are supported by the observation that the c.2935C > T mutant transcript was detected by reverse transcription polymerase chain reaction at a much lower level than the wild‐type allele in whole blood and the lymphoblastoid cell line of the proband, confirming nonsense‐mediated mRNA decay. Accordingly, immunoblotting demonstrated that there was approximately half the amount of SMARCA4 protein in the proband's cells as in controls. This study suggests that SMARCA4 constitutional mutations associated with CSS are not necessarily non‐truncating, and that haploinsufficiency may explain milder CSS phenotypes, as previously reported for haploinsufficient ARID1B. In addition, our case supports the dual role of chromatin remodellers in developmental disorders and cancer, as well as the involvement of SMARCA4 in microphthalmia, confirming previous findings in mouse models and the DECIPHER database. Finally, we speculate that mild CSS might be under‐recognized in a proportion of SCCOHT patients harbouring SMARCA4 mutations. © 2017 The Authors. *The Journal of Pathology* published by John Wiley & Sons Ltd on behalf of Pathological Society of Great Britain and Ireland.

## Introduction

Coffin–Siris syndrome (CSS) (OMIM 135900) is a rare and clinically heterogeneous congenital disorder caused by germline mutations in different subunits of the ATP‐dependent SWI/SNF (SWItch/sucrose non‐fermentable) chromatin remodelling complex involved in transcription, lineage specification, and maintenance of stem cell pluripotency. SMARCA4/BRG1, one of the catalytic subunits of the complex, is mutated in a proportion (∼11%) of CSS patients 1, 2, 3, 4, 5, 6. In addition, *SMARCA4* mutations are associated with small‐cell carcinoma of the ovary hypercalcaemic type (SCCOHT), a malignant undifferentiated tumour of the ovary, characterized by a dismal prognosis and poor response to chemotherapy 7. Because of the high frequency of mutation of the SWI/SNF chromatin remodelling complex in all cancer types, an increased cancer risk in CSS patients has been suggeested, but this has never definitively proven, as malignancies have been only very rarely reported in these patients 8.

All *SMARCA4* germline alterations that have been reported until now in CSS patients are non‐truncating (either missense or small in‐frame deletions) clustered within the highly conserved ATPase/helicase domain, thus suggesting dominant‐negative or gain‐of‐function effects 1, 2, 3, 4, 5, 6. In contrast, almost all SCCOHTs are due to biallelic germline and/or somatic inactivating (nonsense or frameshift) mutations causing complete loss of SMARCA4/BRG1 expression 9, 10, 11.

## Materials and methods

We studied a 15‐year‐old female patient of Italian descent, and her healthy relatives, showing peculiar features of CSS as compared with previously reported cases, such as developmental delay, distinctive facial dysmorphisms (e.g. coarse facies, irregular dentition, and abnormal ears), remarkable hirsutism/hypertrichosis, sparse scalp hair, hearing impairment, and hypoplasia of the distal toenails (supplementary material, Figure S1 and Table S1). At age 13 years, she developed SCCOHT (stage IIIC) with severe hypercalcaemia (17.5 mg/dl). The patient was diagnosed by her attending oncologist and clinical geneticist/dysmorphologist. The study was performed in accordance with the Code of Ethics of the University of Pavia, and parental consent was obtained for publication of images of the patient.

## Results

### Germline analysis

**Figure 1:**
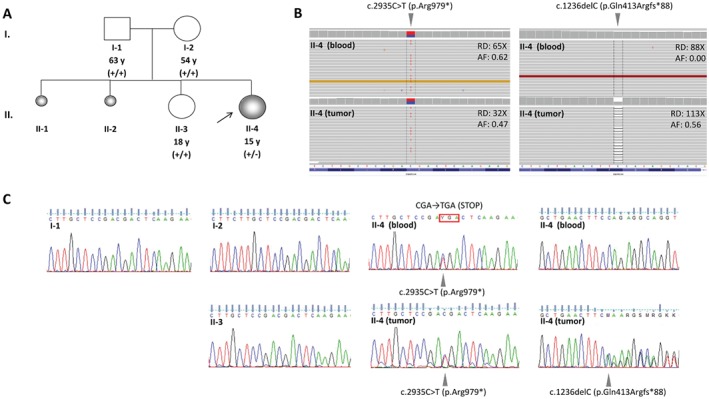
SMARCA4 mutations in the patient's blood and tumour samples. (A) Pedigree of the family. +/−, heterozygous mutation carrier in the germline; +/+, wild‐type in the germline. The c.2935C > T (p.Arg979*) SMARCA4 germline mutation was initially identified by WES in the proband, and relatives were then subjected to targeted Sanger sequencing. (B) Integrative Genomics View (IGV) visualization of the SMARCA4 mutations in the patient's blood and tumour samples. Read depth (RD) and mutation allelic fraction (AF) of both germline and somatic variants are provided. (C) Sanger sequencing analysis in the proband and her relatives. Nucleotide substitutions are indicated by arrowheads.

Conventional karyotyping on peripheral blood metaphases revealed a normal female chromosomal pattern (46,XX), and array comparative genomic hybridization (CGH) and EXCAVATOR did not identify any pathogenic constitutional copy number variation (CNV) (supplementary material, Figure S2). The whole‐exome sequencing (WES) analysis revealed a germline heterozygous nonsense mutation (chr19:11,134,269C > T, GRCh37/hg19; NM_001128849.1:c.2935C > T; NP_001122321.1:p.Arg979*) in exon 19 of *SMARCA4*, which truncated the SMARCA4 protein upstream of both the highly evolutionarily conserved helicase C‐terminal domain (amino acids 1084–1246) and the bromodomain (amino acids 1477–1547). Parental DNA examination revealed that the variant arose *de novo* in the proposita and was absent in the DNA of the 18‐year‐old healthy sister (Figure 1A–C). The c.2935C > T substitution, which is not reported in any of the dbSNP, ExAC, NHLBI ESP and 1000 Genome Project databases, caused the interruption of the reading frame by a premature stop codon, suggesting either a severely truncated translation product or nonsense‐mediated mRNA decay as possible consequences. Interestingly, this variant was previously identified by Ramos *et al*. 9 in the germline DNA of a 9‐year‐old European patient with SCCOHT (stage IA) and hypercalcaemia, but without any reported extratumoural manifestation, whose tumour was not available for investigation of a second somatic hit.

### Somatic analysis

Array CGH and EXCAVATOR analyses excluded the presence of copy number loss in the 19p13.2 region surrounding the *SMARCA4* locus in the formalin‐fixed paraffin‐embedded (FFPE) tumour sample as well as any other causative chromosomal aberration elsewhere, thus confirming the high genomic stability of SCCOHT (supplementary material, Figure S2). By WES, we detected a second somatic frameshift mutation in exon 6 of *SMARCA4* (chr19:11,100,110delC, GRCh37/hg19; NM_001128849.1:c.1236_1236delC; NP_001122321.1:p.Gln413Argfs*88) that caused ablation of the downstream helicase ATP‐binding (amino acids 766–931) and C‐terminal (amino acids 1084–1246) domains, along with the last bromodomain (amino acids 1477–1547) (Figure 1B, C). This 1‐bp deletion, which is not recorded in any database of somatic variants (e.g. COSMIC and ICGC) but has been previously reported by Witkowski *et al*. 7, is expected to trigger nonsense‐mediated decay of the corresponding mRNA transcript, as in the case of the germline mutation. Interestingly, we did not detect any additional pathogenic variant in the SWI/SNF genes or other tumour‐associated genes (supplementary material, Table S2), confirming mutual exclusion of SWI/SNF mutations as well as the peculiar genomic stability of SCCOHT lesions also at the nucleotide level.

**Figure 2:**
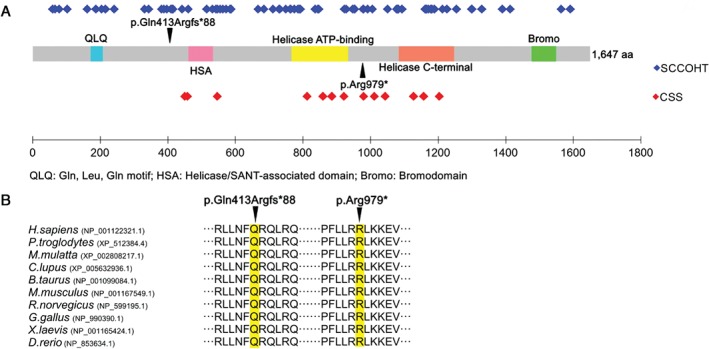
Distribution of SMARCA4 mutations across conserved amino acids. (A) Domain structure of the SMARCA4 protein (UniProtKB‐P51532) composed of 1647 amino acids. Locations of SMARCA4 mutations identified up to now in SCCOHT and CSS patients are represented by blue and red diamonds, respectively (more details are provided in supplementary material, Tables S3 and S5). The two mutated amino acids identified in our patient are indicated by arrowheads. (B) The evolutionary conservation of the mutated amino acids (highlighted in yellow) is shown across 10 different species (from Danio rerio to Homo sapiens), according to NCBI Reference Sequences.

The localization of our two variants across conserved amino acids is in line with previous findings, *SMARCA4* mutations being widely spread along the entire length of the protein in SCCOHT (with the sole exception of the C‐terminal bromodomain), and being clustered within the three central domains (helicase/SANT‐associated domain, helicase ATP‐binding domain, and helicase C‐terminal domain) in CSS (Figure 2; supplementary material, Table S3).

### Functional validation

**Figure 3:**
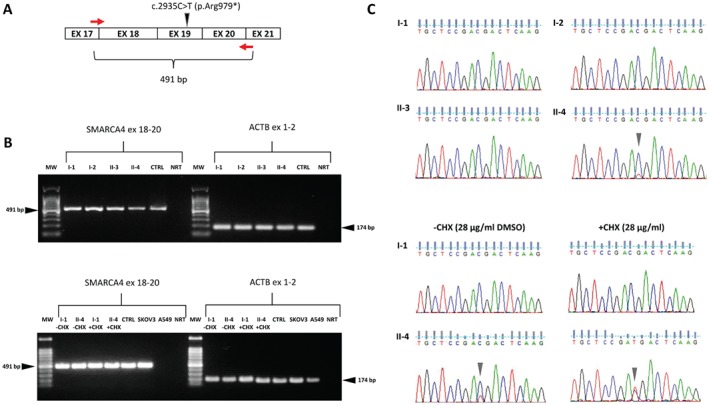
Nonsense‐mediated mRNA decay (NMD) assay in whole blood and B‐LCLs of the patient and relatives. (A) NMD of the mutant transcript was tested by RT‐PCR with specific primers spanning the mutation of interest (c.2935C > T) designed across exon–exon junctions to avoid genomic DNA amplification. (B) Gel electrophoresis of SMARCA4 (491 bp) and housekeeping ACTB (174 bp) cDNA products in whole blood (upper panel) and B‐LCLs treated with 28 μg/ml dimethyl sulphoxide (DMSO) (–CHX) or 28 μg/ml cycloheximide (+CHX) for 4.5 h to suppress NMD (bottom panel). MW: 100‐bp allelic ladder. CTRL: blood cDNA pool of five healthy individuals. NRT: no reverse transcriptase control. SKOV3: human ovarian carcinoma cell line. A549: human lung cancer cell line harbouring a homozygous SMARCA4 nonsense‐inactivating mutation (c.2184_2206del23; p.Gln729Cysfs). I‐1: father. I‐2: mother. II‐3: healthy sister. II‐4: proband. (C) Chromatograms of the germline SMARCA4 c.2935C > T mutation (shown in the forward direction) in whole blood (upper panel) and B‐LCLs (bottom panel). Upper panel: the allele with the c.2935C > T mutation (indicated by an arrowhead) was almost entirely cleared by the NMD mechanism in the whole blood of the patient, with only a residual amount of mutant transcript being detected. As compared with the other relatives, the quantity of the wild‐type allele is approximately decreased in line with the presence of a single copy of the allele. Bottom panel: the mutant allele was rescued by CHX treatment in the patient (II‐4). Note the low concentration of mutant cDNA obtained by RT‐PCR in patient II‐4's immortalized B cells without CHX treatment, confirming the incomplete NMD observed in the whole blood cells.

**Figure 4:**
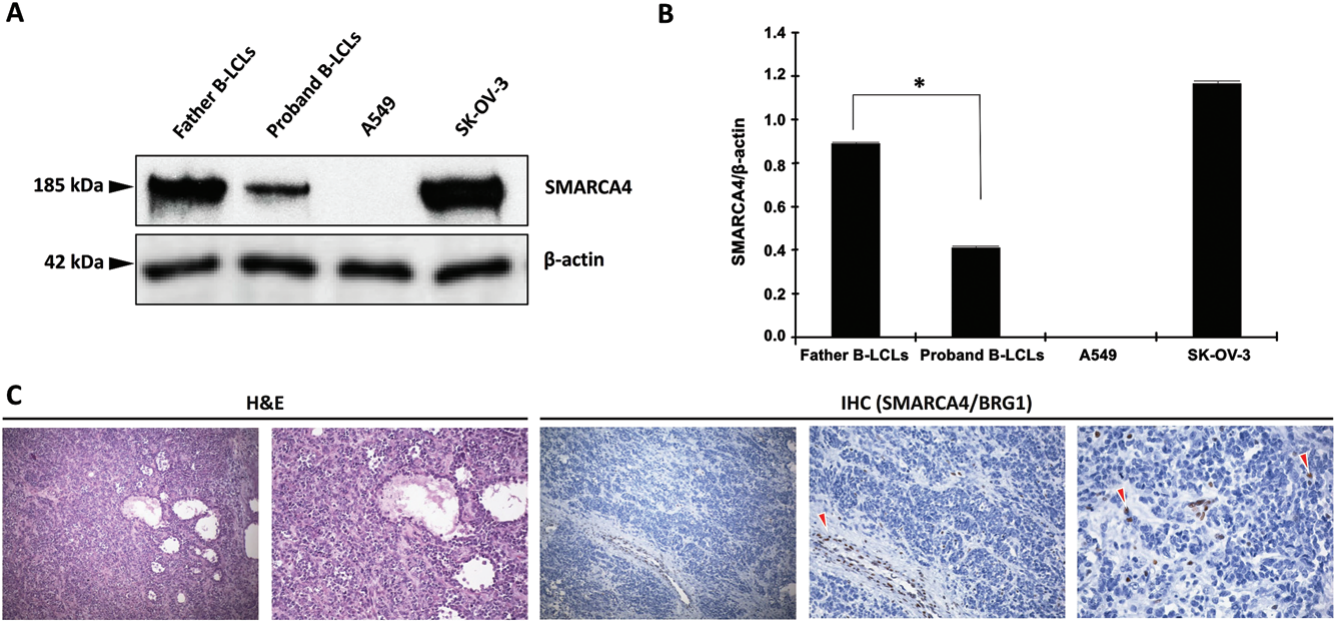
SMARCA4 protein expression in the B‐LCLs and tumour. (A) Immunoblotting with antibody against the N‐terminus of SMARCA4/BRG1 revealed the presence of only the band correlating with the size of the wild‐type full‐length protein product (∼185 kDa). In contrast, the truncated protein, which would result from the interruption of the polypeptide at codon 979 with an expected molecular mass of 108 kDa, was not generated. Protein extracted from the SK‐OV‐3 ovarian adenocarcinoma cell line was used as a positive control. The A549 lung cancer cell line, which harbours homozygous truncating nonsense mutations of SMARCA4 leading to protein loss, served as a negative control. β‐Actin (∼42 kDa) was used as a loading control. Images have been cropped. The figure is representative of three independent experiments. (B) Densitometry analysis of protein bands with ImageJ software. The proband's B‐LCL showed approximately half as much SMARCA4 protein content as the B‐LCL of her unaffected father [mean fold change (father B‐LCLs/proband B‐LCLs): 2.16], as expected, owing to haploinsufficiency. Data were normalized to the level of β‐actin. The asterisk corresponds to statistically significant fold change, *P < 0.0001. (C) Haematoxylin and eosin (H&E) staining, showing a SCCHOT classic‐type histopathological pattern. Magnification: ×10 and ×20, respectively. Immunohistochemistry (IHC) with an antibody against the N‐terminus of SMARCA4/BRG1 showed remarkable loss of protein expression in the SCCHOT FFPE sample. In contrast, internal positive control cells (indicated by red arrowheads) retained intense SMARCA4 nuclear staining. Cell nuclei were counterstained with haematoxylin (blue). Magnification: ×10, ×20, and ×40, respectively.

The c.2935C > T mutant transcript was detected by reverse transcription polymerase chain reaction (RT‐PCR) at a much lower level than the wild‐type allele in the patient's whole blood and B‐lymphoblastoid cell line (B‐LCL). Moreover, cycloheximide (CHX) treatment confirmed that the germline c.2935C > T nonsense mutation resulted in an unstable transcript that was substantially subject to nonsense‐mediated decay (Figure 3). Accordingly, immunoblotting under basal conditions or after CHX stabilizing treatment demonstrated the presence of only the wild‐type full‐length form of the protein (1647 amino acids, ∼184.68 kDa), and conversely, the absence of a possible truncated polypeptide (expected to be composed of 979 amino acids, ∼107.78 kDa) that may theoretically exert a dominant‐negative effect (Figure 4A; supplementary material, Figure S3). The proband's B‐LCL showed half as much protein as the father's B‐LCL and the SK‐OV‐3 control cell line, only one allele being translated in the patient (Figure 4B).

In addition, the immunohistochemical analysis of FFPE tumour tissue revealed tumour‐specific complete loss of SMARCA4 nuclear staining, in keeping with the presence of biallelic inactivating mutations in *SMARCA4*. Notably, as the primary antibody recognizes an N‐terminal epitope located upstream of both p.Arg979* and p.Gln413Argfs*88 truncating mutations (amino acids 200–300), the complete absence of detectable protein expression is consistent with nonsense‐mediated decay of both germline and somatic mutant transcripts (Figure 4C).

### SMARCA4 and microphthalmia

Ophthalmological anomalies have been reported in up to 62% of CSS patients, consisting of severe myopia (up to −18D) or, less often, strabismus, astigmatism, spherophakia, amblyopia, and optic disk coloboma 3. We observed congenital microphthalmia in the proband, a symptom that has never been reported before. Interestingly, it has been demonstrated that *Brg1/Smarca4* regulates both terminal differentiation of lens fibre cells and organized degradation of their nuclei (denucleation) in mouse embryos 12, and a *Brg1* nonsense mutation (Y390X) abrogated retinal cell differentiation in zebrafish 13. Moreover, *N*‐ethyl‐*N*‐nitrosourea‐mutagenized C57BL/6 J mice carrying a mutation in the helicase ATP‐binding domain of *Smarca4* (NM_001174078.1:c.2381C > T; NP_001167549.1:p.Thr794Ile) showed multiple physical defects, including anophthalmia (MGI:5313991), and a *de novo* germline heterozygous deletion of 1.23 Mb spanning *SMARCA4* (chr19:10,640,379‐11,873,382, GRCh37/hg19) has been reported in a female patient with microphthalmos (DECIPHER #250826) (supplementary material, Figures S4 and S5). Surprisingly, this patients showed other clinical features (such as intellectual disability, short toes, recurrent infections, ear abnormalities, and other facial dysmorphisms) highly suggestive of CSS. The deleted region also includes *DOCK6*, which is associated with Adams–Oliver syndrome 2 (OMIM 614219). Although, in rare cases, this condition may include ocular anomalies, such as microphthalmia, retinal detachment, and visual impairment, it is caused by homozygous or, less frequently, compound heterozygous mutations. Importantly, a focused next‐generation sequencing data analysis considering all known microphthalmia‐associated genes failed to identify additional causative mutations in our patient (supplementary material, Table S4 and Figure S6). Taken together, these findings suggest that microphthalmia might be considered to be the result of SMARCA4 deregulation.

## Discussion

We have identified, for the first time, a *SMARCA4* loss‐of‐function mutation, resulting in haploinsufficiency, in a patient with CSS and concomitant early development of SCCOHT (supplementary material, Table S5). There is growing evidence that chromatin remodelling factors may simultaneously cause developmental disorders (with various amounts of intellectual disability) and cancer 14. For instance, frameshift mutations and loss of expression of chromodomain helicase DNA‐binding (CHD) genes have been commonly found in gastric and colorectal cancers 15, and more recently in small‐cell lung cancer 16. Importantly, loss‐of‐function mutations of *CHD7* are also associated with CHARGE syndrome (OMIM 214800), which is characterized, as in the case of CSS, by multiple congenital anomalies, including microphthalmia. Recently, *de novo* missense substitutions in the chromatin remodeller gene *CHD4* have been associated with Sifrim–Hitz–Weiss syndrome (OMIM 617159), and mutations in the same positions have been reported in malignant tumours 17, thus supporting the hypothesis of common alterations shared between intellectual disability syndromes with distinctive dysmorphisms and cancer. Similarly to what has been reported for *ARID1B*‐haploinsufficient individuals, the CSS phenotype in our patient was milder than in all previously reported cases with missense substitutions, which may exert a dominant‐negative effect on other complex proteins. According to this hypothesis, individuals with *SMARCA4* truncating mutations and deletions might share less severe clinical features: in fact, DECIPHER case #303790 with an approximately 247‐kb microdeletion surrounding *SMARCA4* showed mild neurodevelopmental and facial anomalies. Moreover, although further studies are needed, this report broadens the phenotypic spectrum of CSS by including microphthalmia among the clinical features.

In conclusion, our and previous findings suggest that clinical follow‐up should be undertaken in CSS patients to monitor potential neoplasm development and, conversely, that careful investigation of CSS features in patients with SCCOHT should be strongly recommended. In fact, we tentatively speculate that mild CSS might be under‐recognized in a proportion of *SMARCA4‐*positive SCCOHT patients. Furthermore, patients with biallelic somatic mutations but without germline assessment, especially those with an early age of onset, might also harbour constitutional monoallelic alterations, underlying an undiagnosed CSS.

## Author contributions statement

The authors contributed in the following ways: EE: conceived the experiments, interpreted all experimental results, and wrote the paper; NM, AV: participated in the design and interpretation of WES experiments; LDN, BR, SRG: collected clinical data; HDJ: contributed to the set‐up of western blotting experiments and analysis of the results; DV: contributed to the set‐up of WES experiments; PM: performed and analysed immunohistochemical experiments; OZ: conceived the study, supervised all experiments, and performed the final revision of the manuscript. All authors have agreed with the submission in its present form.

**SUPPLEMENTARY MATERIAL ONLINE**

**Supplementary materials and methods**

**Supplementary figure legends**

**Figure S1.** Morphological features of the patient

**Figure S2.** CNVs in patient's blood and tumor samples

**Figure S3.** RT‐PCR and immunoblot assays with recovery after removal of cycloheximide (CHX)

**Figure S4.** Phenotypic effects of chemically‐induced *SMARCA4* mutation (NM_001174078.1:c.2381C>T; NP_001167549.1:p.Thr794Ile) in C57BL/6J mice

**Figure S5.** DECIPHER cases with constitutional CNVs spanning *SMARCA4* (19p13.2) and clinical features resembling microphthalmia

**Figure S6.** Exclusion of additional variants potentially associated with microphthalmia

*Figure S7. RNA‐Seq expression data for *SMARCA4*

**Table S1.** Comparison of clinical features of Coffin‐Siris patients with *SMARCA4* germline mutations

**Table S2.** List of tumor‐associated genes

**Table S3.** *SMARCA4* germline and somatic mutations in individuals with SCCOHT

**Table S4.** List of genes associated with syndromic and isolated microphthalmia

**Table S5.** *SMARCA4* germline mutations in individuals with Coffin‐Siris Syndrome

*Table S6. Summary of metrics of NGS experiments

*Table S7. List of primers used for variant validation and cDNA analysis

*Referred to in supplementary material only

## Supporting information


**Supplementary materials and methods**

**Supplementary figure legends**

**Figure S1. Morphological features of the patient. (A, B)** Pictures of the patient showing peculiar CSS‐associated facial features (coarseness, ptosis, abnormal ears, sparse scalp hair, broad nose, thick eyebrows, long eyelashes, abnormal dentition, large mouth) at 15 years of age. Left microphthalmia is not visible because of ocular prosthesis. **(C)** Picture of the patient at 1 year of age showing microphthalmia. **(D)** Detail of perioral region with remarkable hypertrichosis. **(E, F)** Pictures of the feet showing slightly hypoplastic 5^th^ toenail, hallux valgus, abundant presence of hairs, and persistent toenail onychomycosis.

**Figure S2. CNVs in patient's blood and tumor samples.** Visualization by the EXCAVATOR tool of chromosomes 19p13.2 region corresponding to SMARCA4 locus. Apart from a few polymorphic CNVs, we did not detect any allelic imbalance/LOH in tumor sample (top panel). The matched germline profile (blood) is also shown (bottom panel).

**Figure S3. RT‐PCR and immunoblot assays with recovery after removal of cycloheximide** (**CHX). (A)** Electropherograms of cDNA transcripts derived from father (control) and proband's B‐LCLs after a 1.5 h recovery to restore proteosynthetic activity inhibited by CHX treatment. +CHX: cells treated with 28 μg/ml CHX for 4.5 h; ‐CHX: cells treated with 28 μg/ml DMSO for 4.5 h; +CHX (REC): CHX‐treated cells reincubated into CHX‐free medium for an additional 1.5 h; ‐CHX (REC): DMSO‐treated cells reincubated into CHX‐free medium for an additional 1.5 h. After recovery, a residual amount of mutant transcript (arrowheads) was still detectable, although reduced, in the patient's B‐LCLs. **(B)** Western blot of SMARCA4 in proband (II‐4) and father's (I‐1) CHX‐treated cells before and after recovery, showing that the residual mutant transcript did not generate any truncated protein product in the proband after reversion of CHX treatment. Moreover, the presence of roughly half of SMARCA4 protein in proband compared to father's cells was confirmed by densitometric analysis also after recovery, consistent with haploinsufficiency [Ratio I‐1/II‐4 + CHX: 1.93; Ratio I‐1/II‐4 + CHX (REC): 1.81]. Images have been cropped. The figure is representative of 3 independent experiments.

**Figure S4. Phenotypic effects of chemically‐induced SMARCA4 mutation (NM_001174078.1:c.2381C > T; NP_001167549.1:p.Thr794Ile) in C57BL/6 J mice. (A‐B)** The mutant mouse exhibits anophthalmia, microcephaly, and micrognathia. **(C)** Coronal view of mouse heart by episcopic fluorescence image capture (EFIC) reveals double outlet right ventricle (DORV) and atrioventricular septal defect (AVSD). **(D)** Histological examination of kidney shows abnormal morphology, without evidence of cystic disease. All images have been collected by Professor Cecilia Lo (University of Pittsburgh), on behalf of the Cardiovascular Development Consortium (CvDC), Bench to Bassinet (B2B) Program of the National Heart Lung and Blood Institute (NHLBI).

**Figure S5. DECIPHER cases with constitutional CNVs spanning SMARCA4 (19p13.2) and clinical features resembling microphthalmia.** Patient #250826 clearly manifested microphthalmos, whereas patients #259766 and #269163 presented abnormality of the eyelid and abnormality of the cornea, respectively, possibly underlying microphthalmia. In case #259766, a small heterozygous intragenic deletion of 3.69 Kb (chr19:11,110,404‐11,114,090, GRCh37/hg19) removes SMARCA4 exons 11 and 12, while in case #269163 a 2.33 Mb heterozygous partial duplication at 19p13.2 (chr19:11,101,053‐13,435,131, GRCh37/hg19), including 29 out of 35 SMARCA4 exons, could potentially result in gene disruption and haploinsufficiency. Interestingly, both cases #250826 and #269163 showed other features commonly found in CSS patients. Copy number loss and copy number gain are shown in red and blue color, respectively.

**Figure S6. Exclusion of additional variants potentially associated with microphthalmia.** After NGS data filtering for microphthalmia‐associated genes, we identified two potentially causative genes: RARB and FRAS1, respectively causing microphthalmia syndromic 12 (OMIM 615524) and Fraser syndrome (OMIM 219000), the latter including cryptophthalmos. However, the RARB heretozygous variant was excluded because also present in the unaffected mother, while the compound heterozygous FRAS1 variants were ruled out because: 1) careful clinical re‐evaluation failed to identify any of the Fraiser syndrome major (syndactyly, urinary tract abnormalities, ambiguous genitalia, laryngeal and tracheal anomalies) and minor criteria; and 2) different in silico tools predicted a benign effect for both variants.

**Figure S7. RNA‐Seq expression data for SMARCA4.** The expression levels are higher in EBV‐transformed lymphoblastoid cell lines (green arrowhead) than in whole blood (red arrowhead). The highest SMARCA4 expression is found in testis. RPKM: reads per kilobase per million. Modified from the GTEx Portal (http://www.gtexportal.org).

**Table S1.** Comparison of clinical features of Coffin‐Siris patients with SMARCA4 germline mutations

**Table S2.** List of tumor‐associated genes

**Table S3.** SMARCA4 germline and somatic mutations in individuals with SCCOHT

**Table S4.** List of genes associated with syndromic and isolated microphthalmia

**Table S5.** SMARCA4 germline mutations in individuals with Coffin‐Siris Syndrome

**Table S6.** Summary of metrics of NGS experiments

**Table S7.** List of primers used for variant validation and cDNA analysis
