# Supplementary material for: SMARCA4 inactivating mutations cause concomitant Coffin–Siris syndrome, microphthalmia and small‐cell carcinoma of the ovary hypercalcaemic type
Source: J Pathol. 2017 Jul 25;243(1):9–15. doi: 10.1002/path.4926 (PMC5601212; doi:10.1002/path.4926)
Supplement: Supplementary file 1 — Supplementary materials and methods [file PATH-243-9-s008.doc]

**Supplementary materials and methods**

Reference numbers refer to the main text list

**Clinical report**

The index case, a 15 years old female, was born to healthy Italian non-consanguineous parents. The proband has an older sister and the mother experienced two spontaneous abortions before the first daughter. The second pregnancy was uneventful, even though the ultrasound follow-up showed poor fetal movements and difficulties in evaluating the eyes of the fetus. Delivery occurred at term, with cesarean delivery because of previous section. At birth, weight was 3750 g (79th centile), length was 46 cm (3rd centile) and head circumference (OFC) was reported in the normal range. Physical examination highlighted the presence of congenital left microphthalmia, which required ocular prosthesis since she was 3 months old.

The patient was fed with artificial milk due to impaired lactation and weaning occurred at the 6th month. Acquisition of psychomotor milestones and somatic growth were slightly impaired. She suffered from constipation since the first years of life, sometimes conditioning a rectal prolapse. Isolated episodes of collapse were documented during infancy, due to hypotension and hypoglycemia. Menarche occurred at 11 years followed by regular menstrual cycles, and at the age of 12 she suffered from asthenia and drowsiness for about 3 weeks, in association with recurrent episodes of vomiting and abdominal pain. Blood tests highlighted severe hypercalcemia (17.5 mg/dl; normal range between 8.7 and 10.4 mg/dL) and abdominal ultrasound revealed a rounded mass of 10x9 cm in the pelvis, starting from the left ovary. The further clinical investigation detected suppression of parathormone (PTH), normal values of common serological tumoral markers (AFP, CEA, CA 19.9, CA 15.3, CA 125, hCG), regular renal function and normal electrocardiogram. Abdominal contrast-enhanced CT scan revealed a voluminous mass of 11.5x11x8.5 cm, encapsulated, with non-homogeneous enhancement, in the absence of any pathological adenopathy. CT scan of the head and of the thorax showed no evidence of metastatic proliferation and total body bone scintigraphy failed to detect any abnormal captation. The patient underwent surgical excision of ovarian cancer, and the histological examination of multiple sections diagnosed a small cell ovarian cancer hypercalcemic type (SCCOHT). The architecture was heterogeneous and disordered, with pseudofollicles, hemorrhagic necrosis and strikingly high mitotic index. Moreover, lymphatic invasion occurred at the level of ovarian vessels (stage III C), whereas other metastatic foci were excluded. Immunohistochemical staining showed positivity for WT1, pan-cytokeratin, epithelial membrane antigen (EMA), vimentin and P53 markers; inhibin and placental alkaline phosphatase (PLAP) were negative. The proliferative index (MIB1/Ki67) was estimated above 50%.

After the evaluation of the pediatric oncological reference center, she underwent a total of 6 cycles of chemotherapy (4 with cisplatin, vinblastine, bleomycin, doxorubicin, etoposide, cyclophosphamide and 2 with cisplatin, bleomycin, doxorubicin, etoposide), followed by autologous stem cell transplantation. Imaging examinations at the end of the chemotherapy showed no signs of local relapse, even though small hypodense lesions of the spleen and enlarged para-aortic and inguinal lymph nodes were reported. At the age of 15 years she started hormone replacement therapy with estrogen, due to iatrogenic hypergonadotropic hypogonadism. In fact, she had primary amenorrhea and delayed bone age (13 years), even in the presence of signs of pubertal activation (Tanner stage: B3PH3AH2). A neuropsychological assessment revealed mild intellectual disability with impaired working memory, learning disability, mild speech delay and unusual behavioral manifestations (insecurity and fear). At the last clinical evaluation, she showed many of the peculiar physical characteristics of Coffin-Siris syndrome, such as facial dysmorphisms (coarseness, ptosis, broad nose, abnormal ears with hearing impairment, thick eyebrows, sparse scalp hair), skeletal anomalies (delayed bone age, scoliosis), remarkable hypertrichosis, hypoplastic 5th finger and toenail, hydronephrosis and heart murmurs. The patient did not experience recurrent infections, frequently observed in CSS patients, but persistent onychomycosis (supplementary material, Figure S1). Clinical features of the patient are reported in more detail and compared with previously described cases in supplementary material, Table S1. On physical examination, the proband’s parents and the 18 years old sister were completely healthy. The patient was diagnosed by her attending clinical geneticist/dysmorphologist and oncologist. All subjects provided informed consent to participate in the study and permission to publish pictures of the underage patient. The study was performed in accordance with the Code of Ethics of the University of Pavia.

**Genomic DNA extraction**

We collected peripheral blood samples from all the family members (proband, parents and healthy sister) and the patient’s formalin-fixed paraffin-embedded (FFPE) SCCOHT tumor. Genomic DNA was extracted from blood specimens by using the QIAamp DNA Blood Mini Kit (Qiagen, Hilden, Germany), according to the manufacturer’s instructions. Tumor analysis was performed on the histological sections that had previously been reviewed by a team of experienced gynecological pathologists and selectively dissected from corresponding unstained 5-10 µm slides (2-3 slides each). After deparaffinization, DNA was extracted from the FFPE tissue sections by using the QIAamp DNA FFPE Tissue Kit (Qiagen), following manufacturer’s recommendations. In both cases, DNA was quality-checked and quantified by using the NanoDrop 1000 spectrophotometer (NanoDrop, Wilmington, DE, USA) and the Qubit dsDNA BR Assay kit (Thermo Fisher Scientific, Waltham, MA USA).

**Whole exome sequencing (WES)**

Genomic DNA (approximately 3 µg) extracted from peripheral whole blood was shared through the Covaris S220 focused-ultrasonicator (Covaris, Woburn, MA, USA) to obtain 150-200 bp fragments with treatment time of 360 seconds. Conversely, genomic DNA extracted from FFPE tissues was fragmented under more gentle conditions, with a reduced duration of DNA sharing of 240 seconds. In both cases, quality control on the recovered DNA was performed by using the Bioanalyzer 2100 platform (Agilent Technologies, Santa Clara, CA, USA) to check the size of the fragments. According to the SureSelectXT Target Enrichment Kit (Agilent Technologies), sheared DNA overhangs were end-repaired and purified using the magnetic bead-based Agencourt AMPure XP purification system (Beckman Coulter Genomics, Brea, CA, USA). Then we performed the adding of ‘A’ bases to the 3’ end of the DNA fragments and the ligation of the paired-end adaptors. After a few cycles of PCR amplification (4-6), 750 ng of DNA from the resulting libraries were hybridized to the bait set using the Agilent SureSelect Human All Exon V5 (for the blood analysis) or the Agilent OneSeq Constitutional Research Panel (for the FFPE sample) at 65°C for 24 h. OneSeq protocol was employed in the tumor analysis because, a part from point mutations and indels in clinically relevant genes (according to HGMD, OMIM and ClinVar databases), it simultaneously enables to survey the entire genome for CNVs and copy-neutral LOH (cnLOH), which are frequently detected in tumors. In both cases, following hybrids capture was performed with Streptavidin-coated Dynal magnetic beads (Invitrogen, Carlsbad, CA, USA), according to the manufacturer’s protocol. Captured samples were further purified through the Agencourt AMPure XP beads and subjected to a PCR-based amplification reaction to add specific 6 bp-index tags, accordingly to the SureSelectXT Target Enrichment protocol. Finally, the multiplexed samples were loaded on the Illumina HiSeq 2500 platform (Illumina, San Diego, CA, USA) and analyzed by using a Paired-End 100 bp protocol, according to the Illumina’s instructions. Metrics of NGS experiments are summarized in supplementary material, Table S6.

**Variant filtering**

Briefly, Fastq files were aligned to the reference human genome sequence (GRCh37/hg19) with BWA-MEM and the SAM output file was converted into a sorted BAM file using SAMtools. BAM files underwent local realignment around insertion-deletion sites, duplicate marking and recalibration steps with Genome Analysis Toolkit v1.0.5777. Variant calling was performed with Unified Genotyper, and output VCF files were recalibrated with VariantRecalibrator from GATK v3.6. Genomic variant annotation was carried out with ANNOVAR and only variants with a minimum quality score of 20 and a minimum read depth of 10X were included in the downstream analysis. Thereafter, we excluded the variants reported in 1000 Genomes Project database, the National Heart, Lung and Blood Institute (NHLBI) Exome Sequencing Project (ESP) database, the ExAC (Exome Aggregation Consortium) database, as well as our internal database (composed of approximately 200 individuals), with a population frequency above 5%. We took into account only variants predicted to alter the protein structure or function by at least one of the in silico prediction tools we used (namely Mutation Taster, SIFT and Polyphen-2) and affecting non-synonymous exonic, or splice site (beyond 30 bp of exon/intron boundaries) regions. We further prioritized the candidate variants by using additional bioinformatics tools, such as PredictSNP, Mutation Assessor, VEP, SNPs&Go, PANTHER, PROVEAN, SNAP2, MAPP, and MutPred, and according to amino acid conservation scores. All identified variants were further confirmed by bidirectional Sanger sequencing. Finally, the manual inspection of the Bam files, by using Integrative Genomics Viewer (IGV), allowed us to evaluate the coverage and quality of the aligned reads and, together with the EXCAVATOR tool (https://sourceforge.net/projects/excavatortool/), to identify potential insertions or deletions. Sequencing data were simultaneously analyzed by using the dedicated Agilent’s SureCall software v3.5, especially for CNV and cnLOH detection. Somatic mutations were compared to different databases of known somatic variants, such as COSMIC, ICGC, TCGA, cBioPortal, DriverDBv2, and TumorPortal.

**Sanger sequencing analysis**

PCR reactions were carried out with the AmpliTaq Gold Polymerase Kit (Life Technologies, Foster City, CA, USA) in a final a final volume of 25 μl containing 50 ng of genomic DNA, Gold Buffer 1X, MgCl2 1.5mM, dNTPs 0.2mM, 1U of GoTaq G2 Flexi DNA Polymerase (Promega, Madison, WI, USA) and 10 pmol of both forward and reverse primers specifically designed by using Primer3Plus (supplementary material, Table S7). DNA samples were denatured at 95 °C for 5 min and then amplified for 35 cycles as follows: 95 °C for 30 s, annealing at variable annealing temperature (supplementary material, Table S1) for 30 s, 72 °C for 30 s; and final extension at 72 °C for 7 min. Sequencing reactions were performed using the BigDye Terminator v3.1 Cycle Sequencing Kit on an automated sequencer (3730 DNA Analyzer; Applied Biosystems).

**Conventional karyotyping**

Phytohaemagglutinin (PHA)-stimulated lymphocyte cultures were set up from peripheral blood samples and the chromosomal analysis was carried out on GTG banded metaphases, according to standard procedures.

**Array Comparative Genomic Hybridization (Array-CGH)**

Molecular karyotyping (array-CGH) was performed on DNA samples extracted from patient’s peripheral blood and FFPE tumor samples by using a whole-genome 244K Agilent array (Human Genome CGH Microarray, Agilent Technologies, Santa Clara, CA, USA), according to manufacturer’s protocol. Data were analyzed by using the Agilent Genomic Workbench Standard Edition 6.5.0.58. All genomic positions are reported according to the human genome reference sequence (GRCh37/hg19).

**Immunohistochemical analysis**

Four micrometer thick whole slide sections were prepared from paraffin blocks of formalin-fixed SCCOHT tumor. Unstained FFPE sections were incubated with a rabbit monoclonal antibody specific to SMARCA4/BRG1 (ab110641, 1:25 dilution; Abcam, Cambridge, UK), as previously detailed [9]. The reactions were revealed by using the peroxidase-conjugated biotin-streptavidin complex method (Dako LSAB2 System-HRP; Dako Denmark A/S, Glostrup, Denmark) and 3,3'-diaminobenzidine-tetrahydrochloride-dihydrate (DAB) as chromogenic substrate. Each reaction included a negative control obtained by substituting the primary antibody with dilution buffer. Samples were scored positive if any tumor cell nuclei showed moderate to strong (definite) positive nuclear staining, and negative when tumor cells showed no nuclear staining only if there was adequate nuclear staining of an internal positive control (endothelial cells, fibroblasts or lymphocytes).

**Cell cultures and translation inhibition**

B-lymphoblastoid cell lines (B-LCLs) were generated from heparinized peripheral blood of the proband and her healthy father (control) through Epstein-Barr virus (EBV)-induced transformation. Briefly, PBMCs were incubated with EBV-containing supernatant from the B95.8 cell line (American Type Culture Collection, ATCC, Manassas, VA, US) into T75 flasks containing RPMI 1640 medium (Gibco, Carlsbad, CA, USA) supplemented with 10% heat-inactivated fetal calf serum (FCS; Euroclone, Pero, MI, Italy), 2mM L-glutamine, and 1 µg/ml of cyclosporin A (CsA). Immortalized B lymphocytes were incubated at 37 °C, in a humidified 5% CO_2_ atmosphere for 4 weeks. Each week, 2 ml of culture medium were replaced by complete medium until growth of B-LCL was established.

Drug treatment was carried out by seeding 2x10^6^ cells in complete medium with the addition of 28 µg/ml cycloheximide (CHX; Sigma-Aldrich, Saint Louis, MO, USA) for 4.5 h, in order to stop translation of mRNA and allow the visualization of mRNA species normally subjected to nonsense-mediated decay, as previously described [1,25]. Thereafter, the occurrence of apoptosis, a possible side-effect of CHX treatment, was ruled out through the trypan blue dye exclusion assay of cell viability (Sigma-Aldrich).

A549 (ATCC® CCL-185™) and SK-OV-3 (ATCC® HTB-77™) control cell lines were purchased from the American Type Culture Collection (ATCC, Manassas, VA, US) and maintained using the recommended media and culture conditions. Mutant cell line A549 harbors the homozygous truncating mutation in the exon 15 of *SMARCA4* (c.2184_2206del23; p.Gln729Cysfs), which leads to not detectable or extremely low levels of SMARCA4 protein.

**Analysis of nonsense-mediated mRNA decay (NMD) on whole blood and B-LCLs**

Because *SMARCA4* is supposed to be poorly expressed in human whole blood (supplementary material, Figure S7), RNA is generally isolated from LCLs to test the nonsense mediated decay of the mutant transcript [10]. In this work, we simultaneously analyzed LCLs and whole blood samples in order to compare different (easily accessible) RNA sources, and thus achieve a more detailed representation of the NMD phenomenon.

Peripheral blood was collected in Tempus Blood RNA Tubes (Thermo Fisher Scientific), containing a specific stabilizing reagent that inactivates cellular RNases and selectively precipitates RNA. Before RNA purification, stabilized blood samples were processed as recommended by the manufacturer. RNA was extracted from 3 ml of peripheral blood samples and 2x10^6^ CHX-treated and DMSO-untreated lymphoblastoid cells pelleted for 5 min at 300 x g by using the RNeasy Mini Kit (Qiagen), according to the manufacturer’s specifications and including on-column DNase treatment. After Nanodrop’s quantification and quality-check, 500 ng of purified RNA were reverse-transcribed into single-stranded cDNA using the iScript cDNA Synthesis Kit in a final volume of 20 µl, according to the manufacturer’s instructions (Bio-Rad Laboratories, Hercules, CA, US). Thereafter, cDNA concentration was measured by using the Qubit dsDNA HS Assay kit (Thermo Fisher Scientific). PCR amplification and sequencing of the cDNA region containing the *SMARCA4* germline mutation as well as the house-keeping gene (*ACTB*) were performed with the same conditions used for genomic DNA analysis (supplementary material, Table S1).

**Immunoblot analysis**

Whole-cell extracts were prepared from B-LCLs and control cell lines (A549, SK-OV-3) in RIPA Lysis and Extraction Buffer (Thermo Fisher Scientific), containing protease and phosphatase inhibitors. Total protein concentration was measured by colorimetric assay at 562 nm (Pierce BCA Protein Assay Kit, Thermo Fisher Scientific). Protein lysates (20 µg) were resolved on a 10% SDS-PAGE gel and transferred overnight onto nitrocellulose. After blocking with 5% BSA for 1 h, membranes were probed with rabbit monoclonal primary antibodies specific for SMARCA4/BRG1 (ab110641, 1:1,000 dilution; Abcam) and β-Actin (A5060, 1:2,500 dilution; Sigma-Aldrich), as a loading control. Then, the membranes were incubated with horseradish peroxidase-conjugated goat anti-rabbit secondary polyclonal antibodies (P0448, 1:1,000 dilution; Dako) and proteins were detected through the enhanced chemiluminescence (ECL) system (Pierce ECL Western Blotting Substrate, Thermo Fisher Scientific). Finally, optical density was quantified using ImageJ (https://imagej.nih.gov). Predetermined molecular weights standards were used as markers and protein normalization was performed against β-actin.
