# Supplementary material for: SMARCA4 inactivating mutations cause concomitant Coffin–Siris syndrome, microphthalmia and small‐cell carcinoma of the ovary hypercalcaemic type
Source: J Pathol. 2017 Jul 25;243(1):9–15. doi: 10.1002/path.4926 (PMC5601212; doi:10.1002/path.4926)
Supplement: Supplementary file 2 — Supplementary figure legends [file PATH-243-9-s003.doc]

**Supplementary figure legends**

**Figure S1. Morphological features of the patient.** **(A, B)** Pictures of the patient showing peculiar CSS-associated facial features (coarseness, ptosis, abnormal ears, sparse scalp hair, broad nose, thick eyebrows, long eyelashes, abnormal dentition, large mouth) at 15 years of age. Left microphthalmia is not visible because of ocular prosthesis. **(C)** Picture of the patient at 1 year of age showing microphthalmia. **(D)** Detail of perioral region with remarkable hypertrichosis. **(E, F)** Pictures of the feet showing slightly hypoplastic 5^th^ toenail, hallux valgus, abundant presence of hairs, and persistent toenail onychomycosis.

**Figure S2. CNVs in patient’s blood and tumor samples.** Visualization by the EXCAVATOR tool of chromosomes 19p13.2 region corresponding to *SMARCA4* locus. Apart from a few polymorphic CNVs, we did not detect any allelic imbalance/LOH in tumor sample (top panel). The matched germline profile (blood) is also shown (bottom panel).

**Figure S3. RT-PCR and immunoblot assays with recovery after removal of cycloheximide** (**CHX). (A)** Electropherograms of cDNA transcripts derived from father (control) and proband’s B-LCLs after a 1.5 h recovery to restore proteosynthetic activity inhibited by CHX treatment. +CHX: cells treated with 28 µg/ml CHX for 4.5 h; -CHX: cells treated with 28 µg/ml DMSO for 4.5 h; +CHX (REC): CHX-treated cells reincubated into CHX-free medium for an additional 1.5 h; -CHX (REC): DMSO-treated cells reincubated into CHX-free medium for an additional 1.5 h. After recovery, a residual amount of mutant transcript (arrowheads) was still detectable, although reduced, in the patient’s B-LCLs. **(B)** Western blot of SMARCA4 in proband (II-4) and father’s (I-1) CHX-treated cells before and after recovery, showing that the residual mutant transcript did not generate any truncated protein product in the proband after reversion of CHX treatment. Moreover, the presence of roughly half of SMARCA4 protein in proband compared to father’s cells was confirmed by densitometric analysis also after recovery, consistent with haploinsufficiency [Ratio I-1/II-4 +CHX: 1.93; Ratio I-1/II-4 +CHX (REC): 1.81]. Images have been cropped. The figure is representative of 3 independent experiments.

**Figure S4.** **Phenotypic effects of chemically-induced *SMARCA4* mutation (NM_001174078.1:c.2381C>T; NP_001167549.1:p.Thr794Ile) in C57BL/6J mice. (A-B)** The mutant mouse exhibits anophthalmia, microcephaly, and micrognathia. **(C)** Coronal view of mouse heart by episcopic fluorescence image capture (EFIC) reveals double outlet right ventricle (DORV) and atrioventricular septal defect (AVSD). **(D)** Histological examination of kidney shows abnormal morphology, without evidence of cystic disease. All images have been collected by Professor Cecilia Lo (University of Pittsburgh), on behalf of the Cardiovascular Development Consortium (CvDC), Bench to Bassinet (B2B) Program of the National Heart Lung and Blood Institute (NHLBI).

**Figure S5. DECIPHER cases with constitutional CNVs spanning *SMARCA4* (19p13.2) and clinical features resembling microphthalmia.** Patient #250826 clearly manifested microphthalmos, whereas patients #259766 and #269163 presented abnormality of the eyelid and abnormality of the cornea, respectively, possibly underlying microphthalmia. In case #259766, a small heterozygous intragenic deletion of 3.69 Kb (chr19:11,110,404-11,114,090, GRCh37/hg19) removes *SMARCA4* exons 11 and 12, while in case #269163 a 2.33 Mb heterozygous partial duplication at 19p13.2 (chr19:11,101,053-13,435,131, GRCh37/hg19), including 29 out of 35 *SMARCA4* exons, could potentially result in gene disruption and haploinsufficiency. Interestingly, both cases #250826 and #269163 showed other features commonly found in CSS patients. Copy number loss and copy number gain are shown in red and blue color, respectively.

**Figure S6. Exclusion of additional variants potentially associated with microphthalmia.** After NGS data filtering for microphthalmia-associated genes, we identified two potentially causative genes: *RARB* and *FRAS1*, respectively causing microphthalmia syndromic 12 (OMIM 615524) and Fraser syndrome (OMIM 219000), the latter including cryptophthalmos. However, the *RARB* heretozygous variant was excluded because also present in the unaffected mother, while the compound heterozygous *FRAS1* variants were ruled out because: 1) careful clinical re-evaluation failed to identify any of the Fraiser syndrome major (syndactyly, urinary tract abnormalities, ambiguous genitalia, laryngeal and tracheal anomalies) and minor criteria; and 2) different *in silico* tools predicted a benign effect for both variants.

**Figure S7. RNA-Seq expression data for *SMARCA4*.** The expression levels are higher in EBV-transformed lymphoblastoid cell lines (green arrowhead) than in whole blood (red arrowhead). The highest *SMARCA4* expression is found in testis. RPKM: reads per kilobase per million. Modified from the GTEx Portal (http://www.gtexportal.org).
