# Supplementary material for: SMARCA4 inactivating mutations cause concomitant Coffin–Siris syndrome, microphthalmia and small‐cell carcinoma of the ovary hypercalcaemic type
Source: J Pathol. 2017 Jul 25;243(1):9–15. doi: 10.1002/path.4926 (PMC5601212; doi:10.1002/path.4926)
Supplement: Supplementary file 10 — Table S1. Comparison of clinical features of Coffin‐Siris patients with SMARCA4 germline mutations [file PATH-243-9-s001.doc]

**Supplementary Table S1. Comparison of clinical features of Coffin-Siris patients with *SMARCA4* germline mutations**

| **Clinical Features** | |  | **Present study  (1 patient)** | **Tsurusaki *et al*., 2012 [1] (6 patients)** | **Kosho *et al.,* 2013 [2] (1 patient)** | **Santen *et al*., 2013 [3] (4 patients)** | **Tsurusaki *et al*., 2014 [4] (2 patients)** | **Tzeng *et al*., 2014 [5] (1 patient)** | **Bramswig *et al*., 2015 [6]  (1 patient)** |
| --- | --- | --- | --- | --- | --- | --- | --- | --- | --- |
| Growth | Height | Short stature | + | 80% (4/5) |  | 25% (1/4) | 50% (1/2) | 100% (1/1) |  |
|  | Other | Intrauterine growth retardation (IUGR) |  | 33% (2/6) |  |  | 100% (2/2) | 100% (1/1) |  |
|  |  | Poor overall growth | + |  |  |  |  | 100% (1/1) |  |
| Head and Neck | Head | Microcephaly |  | 80% (4/5) | 100% (1/1) | 25% (1/4) |  | 100% (1/1) | 100% (1/1) |
|  | Face | Coarse facies | + | 100% (6/6) |  |  | 100% (2/2) |  | 100% (1/1) |
|  |  | Low frontal hairline |  |  |  |  |  |  | 100% (1/1) |
|  | Ears | Abnormal ears | + | 83% (5/6) |  | 75% (3/4) | 100% (2/2) | 100% (1/1) | 100% (1/1) |
|  |  | Hearing impairment | + | 50% (3/6) |  |  | 50% (1/2) | 100% (1/1) |  |
|  | Eyes | Visual impairment |  | 83% (5/6) |  |  |  | 100% (1/1) |  |
|  |  | Thick eyebrows | + | 100% (6/6) | 100% (1/1) | 25% (1/4) | 100% (2/2) | 100% (1/1) | 100% (1/1) |
|  |  | Long eyelashes | + | 100% (6/6) | 100% (1/1) | 50% (2/4) | 100% (2/2) | 100% (1/1) | 100% (1/1) |
|  |  | Ptosis | + | 83% (5/6) | 100% (1/1) |  | 50% (1/2) | 100% (1/1) | 100% (1/1) |
|  |  | Congenital microphthalmia | + |  |  |  |  |  |  |
|  | Nose | Flat nasal bridge | + | 66% (4/6) | 100% (1/1) | 25% (1/4) | 100% (2/2) | 100% (1/1) | 100% (1/1) |
|  |  | Broad nose | + | 33% (2/6) | 100% (1/1) |  | 100% (2/2) |  | 100% (1/1) |
|  |  | Anteverted nostrils |  |  |  | 50% (2/4) |  |  | 100% (1/1) |
|  |  | Thick nasal alae |  |  |  | 50% (2/4) |  | 100% (1/1) |  |
|  | Mouth | Wide mouth | + | 50% (3/6) | 100% (1/1) | 75% (3/4) | 100% (2/2) |  | 100% (1/1) |
|  |  | Thin upper lip vermilion | + | 83% (5/6) |  |  | 100% (2/2) | 100% (1/1) | 100% (1/1) |
|  |  | Thick lower lip vermilion | + | 83% (5/6) | 100% (1/1) | 75% (3/4) | 100% (2/2) | 100% (1/1) | 100% (1/1) |
|  |  | Macroglossia |  | 33% (2/6) |  |  |  |  |  |
|  |  | High palate | + | 100% (5/5) |  |  | 100% (2/2) | 100% (1/1) |  |
|  | Philtrum | Short philtrum |  | 50% (3/6) |  |  | 50% (1/2) |  |  |
|  | Teeth | Delayed dentition | + | 60% (3/5) |  | 50% (2/4) |  |  |  |
| Cardiovascular | Heart | Heart defects | + | 33% (2/6) | 100% (1/1) |  |  |  |  |
| Abdomen | Gastro-intestinal | Feeding problems |  | 83% (5/6) |  |  | 100% (2/2) | 100% (1/1) |  |
| Skeletal | Spine | Delayed bone age | + |  |  |  |  |  |  |
|  |  | Scoliosis | + |  |  |  |  | 100% (1/1) |  |
|  | Hands | Hypoplastic to absent terminal phalanges (especially 5th finger) | + | 80% (4/5) |  | 66% (2/3) | 100% (2/2) | 100% (1/1) | 100% (1/1) |
|  |  | Prominent interphalangeal joints | + | 33% (2/6) | 100% (1/1) | 33% (1/3) | 50% (1/2) |  | 100% (1/1) |
|  | Feet | Hypoplastic to absent terminal phalanges (especially 5th toe) | + | 100% (3/3) | 100% (1/1) | 66% (2/3) | 100% (2/2) | 100% (1/1) | 100% (1/1) |
| Skin, Nails, Hair | Nails | Hypoplastic or absent nails | + | 100% (6/6) | 100% (1/1) | 100% (4/4) | 100% (2/2) | 100% (1/1) | 100% (1/1) |
|  | Hair | Hypertrichosis | + | 100% (6/6) | 100% (1/1) | 100% (4/4) | 100% (2/2) |  |  |
|  |  | Sparse scalp hair | + | 50% (3/6) |  | 25% (1/4) | 50% (1/2) |  | 100% (1/1) |
| Neurologic | Central Nervous System | Delayed psychomotor development | + | 100% (6/6) | 100% (1/1) | 100% (4/4) | 100% (2/2) | 100% (1/1) |  |
|  |  | Speech delay | + |  | 100% (1/1) | 100% (4/4) |  | 100% (1/1) |  |
|  |  | Intellectual disability | + |  | 100% (1/1) | 100% (4/4) |  |  |  |
|  |  | Hypotonia |  | 66% (4/6) | 100% (1/1) | 75% (3/4) | 50% (1/2) | 100% (1/1) | 100% (1/1) |
|  |  | Abnormal corpus callosum |  | 100% (1/1) |  | 100% (2/2) | 50% (1/2) | 100% (1/1) | 100% (1/1) |
|  |  | Seizures |  | 33% (2/6) |  |  |  |  |  |
|  | Behavior | Behavioral anomalies | + |  |  |  |  | 100% (1/1) | 100% (1/1) |
| Others |  | Recurrent infections |  | 66% (4/6) | 100% (1/1) | 100% (3/3) | 50% (1/2) |  |  |
|  |  | Inguinal hernia |  | 33% (2/6) | 100% (1/1) |  | 50% (1/2) |  | 100% (1/1) |
|  |  | Hydronephrosis | + |  |  |  |  |  |  |

Reference numbers refer to the main text list
