# Supplementary material for: SMARCA4 inactivating mutations cause concomitant Coffin–Siris syndrome, microphthalmia and small‐cell carcinoma of the ovary hypercalcaemic type
Source: J Pathol. 2017 Jul 25;243(1):9–15. doi: 10.1002/path.4926 (PMC5601212; doi:10.1002/path.4926)
Supplement: Supplementary file 11 — Table S2. List of tumor‐associated genes [file PATH-243-9-s012.doc]

Supplementary Table S2. List of tumor-associated genes

| ABCA4 | CASC5 | DKC1 | FLNA | JAK2 | MSH2 | PDGFRA | RPL5 | TCL1A |
| --- | --- | --- | --- | --- | --- | --- | --- | --- |
| ABCB11 | CASP10 | DNM2 | FLT3 | JAK3 | MSH6 | PDGFRB | RPN1 | TCL6 |
| ABCD1 | CASP8 | DNMT3A | FNBP1 | JAZF1 | MSI2 | PER1 | RPS17 | TERT |
| ABI1 | CBFA2T3 | DOCK8 | FOXL2 | JUN | MSMB | **PHF6** | RPS19 | TET1 |
| ABL1 | CBFB | DUSP10 | FOXO1 | KAT6A | MSN | PHOX2B | RPS24 | TET2 |
| ABL2 | CBL | DUX4L1 | FOXO3 | KAT6B | MSR1 | PICALM | RPS7 | TFE3 |
| ACKR3 | CBLB | EBF1 | FOXO4 | KDM5A | MTAP | PIK3CA | RSPO1 | TFG |
| ACSL3 | CBLC | ECT2L | FOXP1 | KDM5C | MTCP1 | PIK3R1 | RTEL1 | TFPT |
| ACSL6 | CCDC6 | EGFR | FRG1 | KDM6A | MUC1 | PIM1 | RUNX1 | TFRC |
| AFF1 | CCNB1IP1 | EHBP1 | FRG2 | KDR | MUTYH | PINK1 | SBDS | TGFBR1 |
| AFF3 | CCND1 | EIF3H | FSTL3 | KDR | MYB | PLAG1 | SCG5 | TGFBR1 |
| AFF4 | CCND2 | EIF4A2 | FUBP1 | KDSR | MYC | PMEL | SCN9A | TGFBR2 |
| AIP | CCND3 | ELANE | FUS | KIAA1549 | MYCL | PML | SDC4 | THRAP3 |
| AKAP9 | CCNE1 | ELF4 | G6PC3 | KIF1B | MYCN | PMS1 | SDHA | TINF2 |
| AKT1 | CD117 | ELK4 | GALNT12 | KIF5B | MYD88 | PMS2 | SDHAF2 | TLX1 |
| AKT2 | CD274 | ELL | GAR1 | KIT | MYNN | POLD1 | SDHB | TLX3 |
| ALDH2 | CD74 | ELN | GAS7 | KIT | NACA | POLD3 | SDHC | TMC6 |
| ALK | CD79A | EML4 | GATA1 | KLF6 | NBN | POLE | SDHD | TMC8 |
| ALK3 | CD79B | EP300 | GATA2 | KLHDC8B | NBN | POLH | SEPT5 | TMEM127 |
| ALK5 | CD96 | EPCAM | GATA3 | KLK2 | NBS1 | POT1 | SEPT6 | TMPRSS2 |
| AMER1 | CDC25A | EPHB2 | GBA | KMT2A | NCKIPSD | POU2AF1 | SEPT9 | TNFAIP3 |
| AML1 | CDC73 | EPS15 | GJB2 | KMT2C | NCOA1 | POU5F1 | SERPINA1 | TNFRSF14 |
| ANTXR1 | CDH1 | ERBB2 | GLI3 | KMT2D | NCOA2 | POU6F2 | SET | TNFRSF17 |
| ANTXR2 | CDH11 | ERCC1 | GLMN | KRAS | NCOA4 | PPARG | SETD2 | TNFRSF6 |
| APC | CDK12 | ERCC2 | GMPS | KTN1 | NDRG1 | PPM1D | SF3B1 | TOP1 |
| ARHGAP26 | CDK4 | ERCC2 | GNA11 | LAMA5 | NDUFA13 | PPP2R1A | SFPQ | TP53 |
| ARHGEF12 | CDK6 | ERCC3 | GNAQ | LASP1 | NF1 | PRCC | SH2B3 | TP53 |
| **ARID1A** | CDKN1A | ERCC3 | GNAS | LCK | NF2 | PRDM1 | SH2D1A | TPM3 |
| **ARID1B** | CDKN1B | ERCC4 | GOLGA5 | LCP1 | NFE2L2 | PRDM16 | SH3GL1 | TPM4 |
| **ARID2** | CDKN1B | ERCC5 | GOPC | LHFP | NFIB | PRF1 | SHOC2 | TPR |
| **ARID5B** | CDKN1C | ERCC6 | GPC3 | LIFR | NFKB2 | PRKAR1A | SHROOM2 | TRA |
| ARNT | CDKN2A | ERG | GPHN | LIG4 | NHP2 | PRRX1 | SLC25A13 | TRB |
| ASCC1 | CDKN2A | ESCO2 | GREM1 | LKB1 | NIN | PRSS1 | SLC34A2 | TRD |
| ASPSCR1 | CDKN2C | ETV1 | H3F3A | LMO1 | NKX2-1 | PSIP1 | SLC45A3 | TRG |
| ASXL1 | CDX2 | ETV4 | HAX1 | LMO2 | NONO | PTCH1 | SLX4 | TRIM24 |
| ATIC | CEBPA | ETV5 | HERPUD1 | LPP | NOP10 | PTCH2 | SMAD4 | TRIM27 |
| ATM | CHCHD7 | ETV6 | HEY1 | LRIG3 | NOTCH1 | PTEN | SMAD7 | TRIM33 |
| ATR | CHEK2 | EWSR1 | HFE | LYL1 | NOTCH2 | PTPN11 | **SMARCA4** | TRIM37 |
| ATRX | CHEK2 | EXT1 | HIP1 | LYST | NPAT | RAB27A | **SMARCB1** | TRIP11 |
| AXIN2 | CHIC2 | EXT2 | HIST1H4I | MAF | NPM1 | RABEP1 | **SMARCE1** | TSC1 |
| BAP1 | CHN1 | EZH2 | HLF | MAFB | NR4A3 | RAD50 | SMC1A | TSC2 |
| BARD1 | CIC | EZR | HMBS | MALAT1 | NRAS | RAD51B | SMO | TSHR |
| BCL10 | CIITA | FAH | HMGA1 | MALT1 | NSD1 | RAD51B | SNX29 | TTL |
| **BCL11A** | CLP1 | FAM175A | HMGA2 | MAML2 | NTRK1 | RAD51C | SOCS1 | TYK2 |
| **BCL11B** | CLTC | FAM46C | HMGN2P46 | MAP2K1 | NTRK3 | RAD51D | SOS1 | U2AF1 |
| BCL2 | CLTCL1 | FANCA | HNRNPA2B1 | MAP2K2 | NUMA1 | RAD51L1 | SOX2 | UNC13D |
| BCL3 | CNBP | FANCB | HOOK3 | MAP2K4 | NUP214 | RAD51L3 | SPECC1 | UROD |
| BCL6 | CNTRL | FANCB | HOXA11 | MAX | NUP98 | RAD53 | SPRED1 | USP6 |
| **BCL7A** | COL1A1 | FANCC | HOXA13 | MC1R | NUTM1 | RAF1 | SRGAP3 | VEGFR3 |
| BCL9 | COL7A1 | FANCD2 | HOXA9 | MDM2 | NUTM2A | RAG1 | SRSF2 | VHL |
| BCOR | COLCA2 | FANCE | HOXB13 | MDM4 | NUTM2B | RAG2 | SRSF3 | VTI1A |
| BCR | COX6C | FANCF | HOXC11 | MDS2 | OLIG2 | RALGDS | SRY | WAS |
| BIRC3 | CREB1 | FANCG | HOXC13 | MECOM | OMD | RANBP17 | **SS18** | WHSC1 |
| BLM | CREB3L1 | FANCI | HOXD11 | MED12 | OTX2 | RAP1GDS1 | SS18L1 | WIF1 |
| BMP4 | CREB3L2 | FANCL | HOXD13 | MEN1 | p16INK4 | RARA | SSX1 | WRAP53 |
| BMPR1A | CREBBP | FANCM | HRAS | MET | p27KIP1 | RB1 | SSX2 | WRN |
| BMPR1A | CRLF2 | FAP | HSP90AA1 | MFN2 | P2RY8 | RBM15 | SSX4 | WT1 |
| BRAF | CRTC1 | FAS | HSP90AB1 | MITF | PALB2 | RBM8A | STAT3 | WWTR1 |
| BRCA1 | CRTC3 | FBXO11 | IDH1 | MKL1 | PALLD | RECQL4 | STK11 | XPA |
| BRCA2 | CTNNB1 | FBXW7 | IDH2 | MLF1 | PATZ1 | REL | STX11 | XPB |
| BRD3 | CYLD | FCGR2B | IGF2R | MLH1 | PAX3 | RET | STXBP2 | XPC |
| BRD4 | CYP21A2 | FCRL4 | IGH | MLH3 | PAX5 | RHBDF2 | SUFU | XPD |
| BRIP1 | DAXX | FEV | IGK | MLLT1 | PAX7 | RHOH | SUZ12 | XPO1 |
| BTBD12 | DDB2 | FGFR1 | IGL | MLLT10 | PAX8 | RHPN2 | SYK | XRCC2 |
| BTG1 | DDIT3 | FGFR1OP | IKZF1 | MLLT11 | **PBRM1** | RMI2 | TAF15 | XRCC3 |
| BTK | DDX10 | FGFR2 | IL2 | MLLT3 | PBX1 | RMRP | TAL1 | YWHAE |
| BUB1B | DDX5 | FGFR3 | IL21R | MLLT4 | PCM1 | RNASEL | TAL2 | ZBTB16 |
| C2orf44 | DDX6 | FH | IL6ST | MLLT6 | PCSK7 | RNF213 | TCEA1 | ZMYM2 |
| CAMTA1 | DEK | FHIT | IL7R | MN1 | PDCD1LG2 | ROS1 | TCF12 | ZNF331 |
| CANT1 | DICER1 | FIP1L1 | IRF4 | MNX1 | PDE11A | RPL11 | TCF3 | ZNF384 |
| CARD11 | DIP2B | FLCN | ITK | MPL | PDE4DIP | RPL22 | TCF7L1 | ZNF521 |
| CARS | DIS3L2 | FLI1 | JAK1 | MRE11A | PDGFB | RPL35A | TCF7L2 | ZRSR2 |

SWI/SNF genes are shown in bold
