# Supplementary material for: SMARCA4 inactivating mutations cause concomitant Coffin–Siris syndrome, microphthalmia and small‐cell carcinoma of the ovary hypercalcaemic type
Source: J Pathol. 2017 Jul 25;243(1):9–15. doi: 10.1002/path.4926 (PMC5601212; doi:10.1002/path.4926)
Supplement: Supplementary file 12 — Table S3. SMARCA4 germline and somatic mutations in individuals with SCCOHT [file PATH-243-9-s013.doc]

**Supplementary Table S3. *SMARCA4*** germline and somatic mutations in individuals with SCCOHT

| **Reference** | **Patient ID** | **Sample** | **Nucleotide change** | **Amino acid change** | **Mutation type** | **Protein domain** | **Inheritance** | **SMARCA4 expression** |
| --- | --- | --- | --- | --- | --- | --- | --- | --- |
| Kupryjańczyk *et al*. 2013 [18] | PJK1233 | Somatic | c.3277C>T | p.Arg1093* | Nonsense | Helicase  C-terminal |  | Negative |
|  |  | Somatic | c.2184_2206delGCAGTCCTACTATGCCGTGGCCC | p.Gln729Cysfs*4 | Frameshift |  |  |  |
| Kupryjańczyk *et al*. 2013 [18], Moes-Sosnowska  *et al.* 2015 [19] | PJK1182 [18], Proband 1 [19] | Germline | c.3760G>T | p.Glu1254* | Nonsense |  | Paternal | Negative |
|  |  | Somatic | Homozygous | Homozygous | LOH |  |  |  |
| Moes-Sosnowska  *et al.* 2015 | Proband 2 | Germline | c.2351_2352insG | p.Lys785Glufs*39 | Frameshift | Helicase ATP-binding |  | Negative |
| [19] |  | Somatic | Homozygous | Homozygous | LOH |  |  |  |
| Ramos *et al*., 2014 [9] | SCCO-002 | Somatic | c.1237C>T | p.Gln413* | Nonsense |  |  | Negative |
|  |  | Somatic | c.2783C>T | p.Leu928Pro | Missense | Helicase  C-terminal |  |  |
|  | SCCO-008 | Germline | c.2935C>T | p.Arg979* | Nonsense |  |  | na |
|  | SCCO-014 | Somatic | c.2001_2001delG | p.Glu668Argfs*106 | Frameshift |  |  | na |
|  |  | Somatic | c.3481_3481delC | p.Leu1161Serfs*3 | Frameshift | Helicase  C-terminal |  |  |
|  | SCCO-015 | Somatic | c.3565C>T | p.Arg1189* | Nonsense | Helicase  C-terminal |  | na |
|  | SCCO-017 | Germline | c.722_735delGTCCCGGCCCGGCA | p.Gly241fs*41 | Frameshift |  |  | Negative |
|  |  | Somatic | Homozygous | Homozygous | LOH |  |  |  |
|  | DAH23 | Somatic | c.2438+1_2438+2insTGA |  | Splice site | Helicase ATP-binding |  | Negative |
|  | DAH457 | Somatic | c.3277C>T | p.Arg1093* | Nonsense | Helicase  C-terminal |  | na |
|  | DG1006 | Somatic | c.2855_2855delA | p.Lys953Argfs*4 | Frameshift |  |  | Negative |
|  |  | Somatic | c.4771_4774delAGCG | p.Ser1591Cysfs*36 | Frameshift |  |  |  |
|  | DG1219 | Somatic | c.3168+1G>A |  | Splice site |  |  | Negative |
| Ramos *et al*., 2014 [9] | BIN-67  (cell line) | Somatic | c.2438+1G>A |  | Splice site | Helicase ATP-binding |  | Negative |
|  |  | Somatic | c.2439-2A>T |  | Splice site | Helicase ATP-binding |  |  |
| Ramos *et al*., 2014 [20] | SCCO-001 | Somatic | c.482C>T | p.Ala161Val | Missense |  |  | Negative |
|  |  | Somatic | c.1595_1595delC | p.Ala532Valfs*81 | Frameshift | HSA |  |  |
|  | SCCO-004 | Somatic | c.613_613delC | p.Gln205Argfs*98 | Frameshift | QLQ |  | Negative |
|  | SCCO-005 | Somatic | c.3896_3907delGACGAGACCGTCA | p.Glu1300_1303delGlu-Thr-Val-Asn | Inframe deletion |  |  | na |
|  | SCCO-006 | Somatic | c.2293_2315del CTGGTGTCCCTGTACAACAACAA | p.Leu765Profs*51 | Frameshift |  |  | Negative |
|  |  | Somatic | c.2506G>T | p.Gly836* | Nonsense | Helicase ATP-binding |  |  |
|  | SCCO-007 | Somatic | c.991C>T | p.Gln331* | Nonsense |  |  | Negative |
|  |  | Somatic | c.1626_1626delC | p.Ile542Metfs*71 | Frameshift |  |  |  |
|  | SCCO-009 | Somatic | c.3150_3150delC | p.Tyr1050* | Nonsense |  |  | Negative |
|  | SCCO-011 | Somatic | Homozygous  c.3565C>T | Homozygous p.Arg1189* | Nonsense | Helicase  C-terminal |  | Negative |
|  | SCCO-016 | Somatic | Homozygous  c.3985_3986insC | Homozygous p.Arg1329Profs*32 | Frameshift |  |  | Negative |
|  | SCCO-019 | Somatic | c.2531_2532delTT | p.Phe844Cysfs*100 | Frameshift | Helicase ATP-binding |  | Negative |
|  | SCCOHT-1  (cell line) | Somatic | c.3229C>T | p.Arg1077* | Nonsense |  |  | Negative |
|  |  | Somatic | c.3539_3539delC | p.Pro1180Leufs*36 | Frameshift | Helicase  C-terminal |  |  |
| Jelinic *et al*., 2014 | 101 | Somatic | c.3546+1G>A | p.Gln1182_splice | Splice site | Helicase  C-terminal |  | Negative |
| [11] | 102 | Somatic | c.4170+1G>A | p.Lys1390_splice | Splice site |  |  | Negative |
|  | 103 | Somatic | Homozygous  c.3547_3951del | Inframe homozygous  deletion ex 25-26 | Deletion | Helicase  C-terminal |  | Positive |
|  | 104 | Somatic | c.1761+1G>A | p.Lys587_splice | Splice site |  |  | na |
|  |  | Somatic | c.2932C>T | p.Arg978* | Nonsense |  |  |  |
|  | 105 | Somatic | c.991C>T | p.Gln331* | Nonsense |  |  | na |
|  |  | Somatic | c.1626_1626delC | p.Ile542Metfs*71 | Frameshift |  |  |  |
|  | 106 | Somatic | c.1165_1165delC | p.Ser391Profs*20 | Frameshift |  |  | Negative |
| Jelinic *et al*., 2014 [11] | 106 | Somatic | c.3277C>T | p.Arg1093* | Nonsense | Helicase  C-terminal |  |  |
|  | 107 | Somatic | c.3896_3907delGACGAGACCGTCA | p.Glu1300_1303delGlu-Thr-Val-Asn | Inframe deletion |  |  | na |
|  | 108 | Somatic | c.2539C>T | p.Gln847* | Nonsense | Helicase ATP-binding |  | Negative |
|  | 109 | Somatic | c.2287_2288insG | p.Glu763Glyfs*61 | Frameshift |  |  | Negative |
|  |  | Somatic | c.2506G>T | p.Gly836* | Nonsense | Helicase ATP-binding |  |  |
|  | 110 | Somatic | c.3496C>T | p.Gln1166* | Nonsense | Helicase  C-terminal |  | Negative |
|  | 111 | Germline | c.3013C>T | p.Arg1005* | Nonsense |  |  | Negative |
|  |  | Somatic | Homozygous | Homozygous | LOH |  |  |  |
|  | 112 | Somatic | c.2859+1G>A | p.Lys953_splice | Splice site |  |  | Equivocal |
| Jelinic *et al*., 2016 | 113 | Somatic | c.1245+1G>T | p.Gln415_splice | Splice site |  |  | Negative |
| [21] | 114 | Somatic | c.2973+1G>A | p.Lys991_splice | Splice site |  |  | Negative |
| Bailey *et al*., 2015 |  | Somatic | c.1757_1757delA | p.Lys586fs*27 | Frameshift |  |  | Negative |
| [22] |  | Somatic | Homozygous | Homozygous | LOH |  |  |  |
| Le Loarer *et al*., 2015 | SCCOHT2 | Somatic | c.4236_4237delGC | p.Arg1413Glnfs*41 | Frameshift |  |  | Negative |
| [23] |  | Somatic | Homozygous | Homozygous | LOH |  |  |  |
|  | SCCOHT3 | Somatic | c.3216-1G>T |  | Splice site |  |  | Negative |
|  | SCCOHT4 | Somatic | c.2322_2322delC | p.Asn774Lysfs*57 | Frameshift | Helicase ATP-binding |  | Negative |
|  | SCCOHT5 | Somatic | c.3241A>G | p.Lys1081Glu | Missense |  |  | Negative |
|  | SCCOHT6 | Somatic | c.1542C>T | p.Gln515* | Nonsense | HSA |  | Negative |
|  | SCCOHT7 | Somatic | c.3229C>T | p.Arg1077* | Nonsense |  |  | Negative |
|  | SCCOHT8 | Somatic | c.3216-1G>T |  | Splice site |  |  | Negative |
|  | SCCOHT8 | Somatic | c.3229C>T | p.Arg1077* | Nonsense |  |  | Negative |
|  | SCCOHT10 | Somatic | c.3951-1G>T |  | Splice site |  |  | Negative |
| Rabinovich *et al*., 2015 |  | Germline | c.1641_1641delC | p.Asp547Glufs*66 | Frameshift |  |  | Negative |
| [24] |  | Somatic | c.1714A>T | p.Lys572* | Nonsense |  |  |  |
| Witkowski *et al*., 2013 | Mother | Germline | c.3533G>A | p.Trp1178* | Nonsense | Helicase  C-terminal | *De novo* | Negative |
| [25] |  | Somatic | c.2438+1G>T |  | Splice site |  |  |  |
| Witkowski *et al*., 2014 | FA1a | Germline | c.4071+1G>A |  | Splice site |  |  | Negative |
| [10] |  | Somatic | c.1027_1027delG | p.Val343Cysfs*68 | Frameshift |  |  |  |
|  | FA1b | Germline | c.4071+1G>A |  | Splice site |  | Maternal (FA1a) | Negative |
|  |  | Somatic | Homozygous |  | LOH |  |  |  |
|  | FA2a | Germline | c.643C>T | p.Gln215* | Nonsense |  | Paternal | Negative |
|  | FA2b | Germline | c.643C>T | p.Gln215* | Nonsense |  | Maternal (FA2a) | Negative |
|  |  | Somatic | c.1687_1700delAACCTCACGGAGCT | p.Asn563Glyfs*82 | Frameshift |  |  |  |
|  | FA3a | Germline | c.2617-3C>G |  | Splice site | Helicase ATP-binding | Parent (unknown) | Negative |
|  | FA3b | Germline | c.2617-3C>G |  | Splice site | Helicase ATP-binding | Maternal (FA3a) | Negative |
|  |  | Somatic | Homozygous |  | LOH |  |  |  |
|  | FA4a | Germline | c.3239G>A | p.Gly1080Asp | Missense |  |  | Positive |
|  |  | Somatic | Homozygous | Homozygous | LOH |  |  |  |
|  | FA4b | Germline | c.3239G>A | p.Gly1080Asp | Missense |  | Maternal (FA4a) | Negative |
|  |  | Somatic | c.1326_1326delC | p.Ser442Argfs*59 | Frameshift |  |  |  |
|  | NF1 | Germline | c.1224_1226delGCTinsAG | p.Leu409Glyfs*2 | Frameshift |  |  | Negative |
|  | NF2 | Germline | c.1663C>T | p.Gln555* | Nonsense |  |  | Negative |
|  |  | Somatic | Homozygous | Homozygous | LOH |  |  |  |
|  | NF3 | Somatic | Homozygous  c.3496C>T | Homozygous p.Gln1166* | Nonsense | Helicase  C-terminal |  | Negative |
|  | NF4 | Germline | c.3638_3638delA | p.Lys1213Argfs*3 | Frameshift | Helicase  C-terminal |  | Negative |
|  | NF5 | Germline | c.3480_3481insG | p.Leu1161Alafs*15 | Frameshift | Helicase  C-terminal |  | Negative |
|  | NF6 | Somatic | c.1378C>T | p.Gln460* | Nonsense | HSA |  | Negative |
|  | NF6 | Somatic | c.2129_2129delC | p.Lys711Serfs*63 | Frameshift |  |  |  |
|  | NF7 | Somatic | c.2245_2246insA | p.Met749Asnfs*75 | Frameshift |  |  | Negative |
| Witkowski *et al.*, 2014 | UN1 | Somatic | c.561C>G | p.Thr187* | Nonsense | QLQ |  | Negative |
| [10] |  | Somatic | c.2362C>T | p.Gln788* | Nonsense | Helicase ATP-binding |  |  |
|  | UN2 | Somatic | c.3676C>T | p.Gln1226* | Nonsense | Helicase  C-terminal |  | Negative |
|  | UN3 | Germline | c.2932C>T | p.Arg978* | Nonsense |  |  | Negative |
|  |  | Somatic | Homozygous | Homozygous | LOH |  |  |  |
|  | UN4 | Somatic | c.3531_3531delC | p.Trp1178Glyfs*38 | Frameshift | Helicase  C-terminal |  | Negative |
|  |  | Somatic | c.4687_4687delG | p.Ile1564Serfs*32 | Frameshift |  |  |  |
|  | UN5 | Somatic | Homozygous c.2275-1G>T |  | Splice site |  |  | Negative |
|  | UN6 | Somatic | Homozygous  c.2838_2838delC | Homozygous p.Phe947Leufs*3 | Frameshift |  |  | Negative |
|  | UN7 | Germline | c.1141C>T | p.Arg381* | Nonsense |  |  | Negative |
|  |  | Somatic | Homozygous | Homozygous | LOH |  |  |  |
|  | UN8 | Somatic | Homozygous  c.2190_2191insG | Homozygous p.Tyr731Valfs*10 | Frameshift |  |  | Negative |
|  | UN9 | Somatic | c.1420+1G>T |  | Splice site |  |  | Positive |
|  | UN10 | Somatic | c.2049_2049delC | p.Val684Trpfs*90 | Frameshift |  |  | Negative |
|  | UN11 | Somatic | c.3244_3244delT | p. Phe1082Leufs*24 | Frameshift |  |  | Negative |
|  | UN12 | Somatic | c.2766G>A | p.Trp922* | Nonsense | Helicase ATP-binding |  | Negative |
|  | UN13 | Somatic | Homozygous  c.3546+1G>T |  | Splice site |  |  | Negative |
|  | UN14 | Somatic | c.2915T>C | p.Leu972Pro | Missense |  |  | Negative |
|  |  | Somatic | c.3168+1G>C |  | Splice site |  |  |  |
|  | UN15 | Somatic | Homozygous c.1761+2T>A |  | Splice site |  |  | Negative |
|  | UN16 | Somatic | Homozygous c.233_237delCCATGinsACC | Homozygous p.Ser78Tyrfs*3 | Frameshift |  |  | Negative |
| Lavrut *et al*., 2016 [26],  Le Loarer *et al*., 2015  [23] | SCCOHT1 [25] | Germline | c.300_301delAG | p.Gly102Profs*26 | Frameshift |  |  | Negative |
|  |  | Somatic | Homozygous | Homozygous | LOH |  |  |  |
| Witkowski *et al.*, 2016 | Family 1 (III:1, III:3) | Germline | c.2859+1G>C |  | Splice site |  | Paternal (II:4) | Negative |
| [27] |  | Somatic | Homozygous |  | LOH |  |  |  |
|  | Family 2 (III:2) | Germline | c.175C>T | p.Asn59* | Nonsense |  | Maternal (II:3) | Negative |
|  |  | Somatic | c.2375T>C | p.Leu792Pro | Missense | Helicase ATP-binding |  |  |
| Witkowski *et al*., 2016 | NF10 | Germline | c.3277C>T | p.Arg1093* | Nonsense | Helicase  C-terminal |  | Negative |
| [7] | NF11 | Somatic | c.1751_1751delA | p.Lys584Argfs*29 | Frameshift |  |  | Negative |
|  | NF12 | Somatic | c.3512_3513delTG | p.Val1171Aspfs*4 | Frameshift | Helicase  C-terminal |  | Negative |
|  |  | Somatic | c.3488_3525delTGAACCTCCAGTCGGCAGACACTGTGATCATTTTTGAC | p.Leu1163Glnfs*67 | Frameshift | Helicase  C-terminal |  |  |
|  | NF13 | Germline | c.3229C>T | p.Arg1077* | Nonsense |  |  | Negative |
|  | NF14 | Somatic | c.2973+1 G>A |  | Splice site |  |  | Negative |
|  | NF15 | Somatic | c.2838_2838delC | p.Phe947Leufs*3 | Frameshift |  |  | Negative |
|  | NF17 | Somatic | c.2274+2T>G |  | Splice site |  |  | Negative |
|  | NF18 | Somatic | c.1189C>T | p.Arg397* | Nonsense |  |  | Negative |
|  |  | Somatic | c.3216-2A>G |  | Splice site |  |  |  |
|  | NF21 | Somatic | c.196C>T | p.Gln66* | Nonsense |  |  | Negative |
|  |  | Somatic | c.2859+1G>C |  | Splice site |  |  |  |
|  | FA7a | Germline | c.2859+1G>C |  | Splice site |  | Paternal | Negative |
|  | FA7b | Germline | c.2859+1G>C |  | Splice site |  | Paternal | Negative |
|  | FA8a | Germline | c.175C>T | p.Gln59* | Nonsense |  |  | Negative |
|  | FA8b | Germline | c.175C>T | p.Gln59* | Nonsense |  | Maternal | Negative |
|  |  | Somatic | c.2375C>T | p.Leu792Pro | Missense | Helicase ATP-binding |  |  |
|  | Case 24 | Somatic | c.1236_1236delC | p.Gln413Argfs*88 | Frameshift |  |  | Negative |
|  | Case 24 | Somatic | c.2970_2970delA | p.Lys991Argfs*28 | Frameshift |  |  |  |
|  | Case 25 | Somatic | c.2123+1G>A |  | Splice site |  |  | Negative |
|  | Case 27 | Somatic | c.1119-1G>C |  | Splice site |  |  | Negative |
| Present study | II-4 | Germline | c.2935C>T | p.Arg979* | Nonsense |  | *De novo* | Negative |
|  |  | Somatic | c.1236_1236delC | p.Gln413Argfs*88 | Frameshift |  |  |  |

na: not available

HSA: Helicase/SANT-associated domain (IPR014012)

QLQ: Gln, Leu, Gln motif (IPR014978)

LOH: loss of heterozygosity
