# Supplementary material for: SMARCA4 inactivating mutations cause concomitant Coffin–Siris syndrome, microphthalmia and small‐cell carcinoma of the ovary hypercalcaemic type
Source: J Pathol. 2017 Jul 25;243(1):9–15. doi: 10.1002/path.4926 (PMC5601212; doi:10.1002/path.4926)
Supplement: Supplementary file 13 — Table S4. List of genes associated with syndromic and isolated microphthalmia [file PATH-243-9-s002.doc]

**Supplementary Table S4. List of genes associated with syndromic and isolated microphthalmia**

| **Gene** | **Location** | **Phenotype** | **OMIM ID** | **Inheritance** |
| --- | --- | --- | --- | --- |
| *RARB* | 3p24.2 | Microphthalmia, syndromic 12 (MCOPS12) | 615524 | AD |
| *SOX2* | 3q26.33 | Microphthalmia, syndromic 3 (MCOPS3) | 206900 | AD |
| *MAB21L2* | 4q31.3 | Microphthalmia, syndromic 14 (MCOPS14) | 615877 | AD, AR |
| *VAX1* | 10q25.3 | Microphthalmia, syndromic 11 (MCOPS11) | 614402 | AR |
| *BMP4* | 14q22.2 | Microphthalmia, syndromic 6 (MCOPS6) | 607932 | AD |
| *OTX2* | 14q22.3 | Microphthalmia, syndromic 5 (MCOPS5) | 610125 | AD |
| *STRA6* | 15q24.1 | Microphthalmia, syndromic 9 (MCOPS9) | 601186 | AR |
| *HCCS* | Xp22.2 | Microphthalmia, syndromic 7 (MCOPS7) | 309801 | XLD |
| *BCOR* | Xp11.4 | Microphthalmia, syndromic 2 (MCOPS2) | 300166 | XLD |
| *HMGB3* | Xq28 | Microphthalmia, syndromic 13 (MCOPS13) | 300915 | XL |
| *NAA10* | Xq28 | Microphthalmia, syndromic 1 (MCOPS1) | 309800 | XL |
| *ABCB6* | 2q35 | Microphthalmia, isolated, with coloboma 7 (MCOPCB7) | 614497 | AD |
| *TENM3* | 4q34.3-q35.1 | Microphthalmia, isolated, with coloboma 9 (MCOPCB9) | 615145 | AR |
| *SHH* | 7q36.3 | Microphthalmia, isolated, with coloboma 5 (MCOPCB5) | 611638 | AD |
| *GDF3* | 12p13.31 | Microphthalmia, isolated 7 (MCOP7) | 613704 | AD |
| *GDF6* | 8q22.1 | Microphthalmia, isolated 4 (MCOP4) | 613094 | AD |
| *CHX10* | 14q24.3 | Microphthalmia, isolated 2 (MCOP2) | 610093 | AR |
| *RBP4* | 10q23.33 | Microphthalmia, isolated, with coloboma 10 (MCOPCB10) | 616428 | AD |
| *PRSS56* | 2q37.1 | Microphthalmia, isolated 6 (MCOP6) | 613517 | AR |
| *MFRP* | 11q23.3 | Microphthalmia, isolated 5 (MCOP5) | 611040 | AR |
| *ALDH1A3* | 15q26.3 | Microphthalmia, isolated 8 (MCOP8) | 615113 | AR |
| *RAX* | 18q21.32 | Microphthalmia, isolated 3 (MCOP3) | 611038 | AR |
| *BMP7* | 20q13.31 | Developmental eye anomalies, developmental delay, deafness, scoliosis, cleft palate | 112267 | AD |
| *SIX6* | 14q23.1 | Optic disc anomalies with retinal and/or macular dystrophy | 606326 | AR |
| *FOXE3* | 1p33 | Aphakia, congenital primary | 610256 | AR |
| *CRYBA4* | 22q12.1 | Cataract 23 (with bilateral microphthalmia) | 610425 | AD |
| *SMOC1* | 14q24.2 | Microphthalmia with limb anomalies (MLA) | 206920 | AR |
| *PITX3* | 10q24.32 | Cataract 11, syndromic | 610623 | AD |
| *CHD7* | 8q12.2 | CHARGE syndrome | 214800 | AD |
| *FREM1* | 9p22.3 | Manitoba oculotrichoanal syndrome (MOTA) | 248450 | AR |
| *COL4A1* | 13q34 | Brain small vessel disease with or without ocular anomalies (BSVD) | 607595 | AD |
| *CYP1B1* | 2p22.2 | Peters anomaly with coloboma/microphthalmia | 604229 | AD, AR  continued |
| *FOXC1* | 6p25.3 | Axenfeld-Rieger syndrome, type 3 (RIEG3) with anophthalmia, microphthalmia, and coloboma | 602482 | AD |
| *FRAS1* | 4q21.21 | Fraser syndrome with cryptophthalmos/microphthalmia/anophthalmia | 219000 | AR |
| *GRIP1* | 12q14.3 | Fraser syndrome with cryptophthalmos/microphthalmia/anophthalmia | 219000 | AR |
| *FREM2* | 13q13.3 | Fraser syndrome with cryptophthalmos/microphthalmia/anophthalmia | 219000 | AR |
| *NDP* | Xp11.3 | Norrie disease | 310600 | XLR |
| *PAX6* | 11p13 | Bilateral microphthalmia, coloboma, primary aphakia, iris hypoplasia, sclerocornea and congenital glaucoma | 607108 | AD |
| *PITX2* | 4q25 | Axenfeld-Rieger syndrome, type 1 (RIEG1) | 180500 | AD |
