# Supplementary material for: SMARCA4 inactivating mutations cause concomitant Coffin–Siris syndrome, microphthalmia and small‐cell carcinoma of the ovary hypercalcaemic type
Source: J Pathol. 2017 Jul 25;243(1):9–15. doi: 10.1002/path.4926 (PMC5601212; doi:10.1002/path.4926)
Supplement: Supplementary file 14 — Table S5. SMARCA4 germline mutations in individuals with Coffin‐Siris Syndrome [file PATH-243-9-s009.doc]

**Supplementary Table S5. *SMARCA4* germline mutations in individuals with Coffin-Siris Syndrome**

| **Reference** | **Patient ID** | **Nucleotide change** | **Aminoacid change** | **Mutation type** | **Protein domain** | **Inheritance** |
| --- | --- | --- | --- | --- | --- | --- |
| Tsurusaki *et al*., 2012 | 9 | c.1636_1638del AAG | p.Lys546del | In-frame deletion |  | *De novo* |
| [1] | 7 | c.2576C>T | p.Thr859Met | Missense | Helicase ATP-binding | *De novo* |
|  | 5 | c.2653C>T | p.Arg885Cys | Missense | Helicase ATP-binding | *De novo* |
|  | 16 | c.2761C>T | p.Leu921Phe | Missense | Helicase ATP-binding | *De novo* |
|  | 25 | c.3032T>C | p.Met1011Thr | Missense |  | *De novo* |
|  | 17 | c.3469C>G | p.Arg1157Gly | Missense | Helicase C-terminal | *De novo* |
| Tsurusaki *et al*., 2014 | 32 | c.1372_1395del | p.Lys458_Glu465del | In-frame deletion |  | *De novo* |
| [4] | 14 | c.2654G>A | p.Arg885His | Missense | Helicase ATP-binding | *De novo* |
| Kosho *et al*., 2013 [2] | 7 | c.2654G>A | p.Arg885His | Missense | Helicase ATP-binding | Unknown |
| Santen *et al*., 2013 | 46 | c.1349C>A | p.Ala450Asp | Missense |  | *De novo* |
| [3] | 70 | c.3127C>T | p.Arg1043Trp | Missense |  | *De novo* |
|  | 42 | c.3380A>G | p.Asp1127Gly | Missense | Helicase C-terminal | Unknown |
|  | 2 | c.3608G>A | p.Arg1203His | Missense | Helicase C-terminal | Unknown |
| Tzeng *et al*., 2014 [5] | Propositus | c.2434C>T | p.Leu812Phe | Missense | Helicase ATP-binding | *De novo* |
| Bramswig *et al*., 2015  [6] | K2430 | chr19:g.11,146,701_11,172,353del |  | Intragenic deletion  (25,652 bp - ex. 30-35) |  | *De novo* |
| Present study | II-4 | c.2935C>T | p.Arg979* | Stop-gain |  | *De novo* |
