# Supplementary material for: SMARCA4 inactivating mutations cause concomitant Coffin–Siris syndrome, microphthalmia and small‐cell carcinoma of the ovary hypercalcaemic type
Source: J Pathol. 2017 Jul 25;243(1):9–15. doi: 10.1002/path.4926 (PMC5601212; doi:10.1002/path.4926)
Supplement: Supplementary file 15 — Table S6. Summary of metrics of NGS experiments [file PATH-243-9-s005.doc]

**Supplementary Table S6. Summary of metrics of NGS experiments**

| **Sample** | **Target** | **Mean read depth** | **% bases > 15** |
| --- | --- | --- | --- |
| **Peripheral blood** | Whole exome | 56.52 | 83.1 |
|  | *SMARCA4* | 62.74 | 90.2 |
| **Tumor (FFPE)** | Whole exome | 118.64 | 84.4 |
|  | *SMARCA4* | 48.50 | 73.5 |
