# Supplementary material for: SMARCA4 inactivating mutations cause concomitant Coffin–Siris syndrome, microphthalmia and small‐cell carcinoma of the ovary hypercalcaemic type
Source: J Pathol. 2017 Jul 25;243(1):9–15. doi: 10.1002/path.4926 (PMC5601212; doi:10.1002/path.4926)
Supplement: Supplementary file 16 — Table S7. List of primers used for variant validation and cDNA analysis [file PATH-243-9-s011.doc]

**Supplementary Table S7. List of primers used for variant validation and cDNA analysis**

| **Primer name** | **Application** | **Sequence (5'->3')** | **Tm (°C)** | **T Ta (°C)** | **Amplicon size (bp)** | **Chomosomal coordinates** |
| --- | --- | --- | --- | --- | --- | --- |
| SMARCA4_ex6_F | Sanger | GGGGCTATCTCCCTCTCTGCT | 66.9 | 60.0 | 361 | chr19:11099822-11100182 (GRCh37/hg19) |
| SMARCA4_ex6_R | validation | CCTTAGCAGCAGTGTTGGTG | 63.5 |  |  |  |
| SMARCA4_ex19_F | Sanger | CCGCACCTTCTAGTGAGACC | 63.8 | 60.0 | 311 | chr19:11134119-11134429 (GRCh37/hg19) |
| SMARCA4_ex19_R | Validation | CCCAAGCTGGATTGACCA | 64.9 |  |  |  |
| SMARCA4_ex18-20_F | cDNA analysis | CACATCCTCGCCAAGATCC | 65.7 | 60.0 | 491 | NM_001128849.1: c.2602-3092 |
| SMARCA4_ex18-20_R |  | CCGCCTTTGCCCTTCTT | 65.2 |  |  |  |
| ACTB_ex1-2_F | cDNA analysis | AGCCTCGCCTTTGCCGA | 69.6 | 60.0 | 174 | NM_001101.3: c.142-315 |
| ACTB_ex1-2_F |  | CTGGTGCCTGGGGCG | 68.4 |  |  |  |
